# Transcriptome and venom proteome of the box jellyfish *Chironex fleckeri*

**DOI:** 10.1186/s12864-015-1568-3

**Published:** 2015-05-27

**Authors:** Diane L Brinkman, Xinying Jia, Jeremy Potriquet, Dhirendra Kumar, Debasis Dash, David Kvaskoff, Jason Mulvenna

**Affiliations:** Australian Institute of Marine Science, Townsville, QLD Australia; Infectious Diseases Program, QIMR Berghofer Medical Research Institute, Brisbane, QLD Australia; G.N. Ramachandran Knowledge Center for Genome Informatics, CSIR-Institute of Genomics and Integrative Biology, New Delhi, India; The University of Queensland Centre for Clinical Research, Royal Brisbane and Women’s Hospital, Brisbane, QLD Australia; The University of Queensland, School of Biomedical Sciences, Brisbane, QLD Australia

**Keywords:** *Chironex fleckeri*, Venom, Transcriptome, Proteome

## Abstract

**Background:**

The box jellyfish, *Chironex fleckeri*, is the largest and most dangerous cubozoan jellyfish to humans. It produces potent and rapid-acting venom and its sting causes severe localized and systemic effects that are potentially life-threatening. In this study, a combined transcriptomic and proteomic approach was used to identify *C. fleckeri* proteins that elicit toxic effects in envenoming.

**Results:**

More than 40,000,000 Illumina reads were used to *de novo* assemble ∼ 34,000 contiguous cDNA sequences and ∼ 20,000 proteins were predicted based on homology searches, protein motifs, gene ontology and biological pathway mapping. More than 170 potential toxin proteins were identified from the transcriptome on the basis of homology to known toxins in publicly available sequence databases. MS/MS analysis of *C. fleckeri* venom identified over 250 proteins, including a subset of the toxins predicted from analysis of the transcriptome. Potential toxins identified using MS/MS included metalloproteinases, an alpha-macroglobulin domain containing protein, two CRISP proteins and a turripeptide-like protease inhibitor. Nine novel examples of a taxonomically restricted family of potent cnidarian pore-forming toxins were also identified. Members of this toxin family are potently haemolytic and cause pain, inflammation, dermonecrosis, cardiovascular collapse and death in experimental animals, suggesting that these toxins are responsible for many of the symptoms of *C. fleckeri* envenomation.

**Conclusions:**

This study provides the first overview of a box jellyfish transcriptome which, coupled with venom proteomics data, enhances our current understanding of box jellyfish venom composition and the molecular structure and function of cnidarian toxins. The generated data represent a useful resource to guide future comparative studies, novel protein/peptide discovery and the development of more effective treatments for jellyfish stings in humans. (Length: 300).

**Electronic supplementary material:**

The online version of this article (doi:10.1186/s12864-015-1568-3) contains supplementary material, which is available to authorized users.

## Background

Box jellyfish (Class Cubozoa) produce venoms that are designed to swiftly incapacitate prey and deter predators, but they also cause adverse effects in envenomed humans. Cubozoan venoms are stored within complex intracellular structures (nematocysts) that are housed within specialized cells (nematocytes) located mainly in the tentacles of the jellyfish. When triggered to discharge, each nematocyst explosively releases a harpoon-like tubule that injects a toxic cocktail of venom components into the victim or prey.

*Chironex fleckeri* is the largest and most venomous box jellyfish species. It inhabits the tropical coastal waters of Australia and is renowned for its ability to inflict extremely painful and potentially life threatening stings to humans. Symptoms of *C. fleckeri* envenoming can include the rapid onset of severe cutaneous pain and inflammation, dermonecrosis, dyspnoea, transient hypertension, hypotension, cardiovascular collapse and cardiac arrest (reviewed in [1]). Due to its clinical importance, *C. fleckeri* has remained one of the most intensively researched box jellyfish species. Over five decades of research on whole or fractionated *C. fleckeri* tentacle extracts and nematocyst-derived venom has established that *C. fleckeri* toxins elicit a diverse range of bioactivities including nociception, *in vitro* cytotoxicity in cultured myocytes (cardiac, skeletal and smooth muscle) and hepatocytes, haemolytic activity and pore formation in mammalian cell membranes, neurotoxicity and myotoxicity in nerve and muscle preparations, and *in vivo* dermonecrotic, cardiovascular and lethal effects in a variety of experimental animals [1-5].

In recent studies, the potent *in vitro* haemolytic and *in vivo* cardiovascular activities of *C. fleckeri* venom have been attributed primarily to the action of a subset of *C. fleckeri* toxins (CfTXs) that are members of a taxonomically restricted family of cnidarian pore-forming toxins [2,5]. A single proteomics study of *C. fleckeri* venom revealed that several isoforms of the CfTXs are highly abundant in the venom proteome [6], but due to the lack of genomic and transcriptomic data for cubozoans, few other potential toxins were identified [6]. However, the diversity of biological activities associated with *C. fleckeri* venom and the complexity of its venom composition, suggest that other biologically important venom components are yet to be identified. These novel cubozoan venoms could represent a source of potentially useful bioactive compounds for the development of novel therapeutics.

Advances in computational techniques for the assembly and annotation of sequence data have enabled the rapid characterization of biologically important protein mixtures from a range of organisms [7,8]. In this work we utilized Illumina sequencing in concert with tandem mass spectroscopy (MS/MS) to conduct a large-scale exploration of the transcriptome and venom proteome of *C. fleckeri*. The newly obtained transcriptomic data facilitated the detection of several new CfTX isoforms and other putative toxin families, including metalloproteinases, that have not been previously identified in cubozoan venoms. This study not only provides extensive information on the molecular diversity of toxins in *C. fleckeri* venom, but also provides the first overview of a box jellyfish transcriptome; thus representing a valuable resource for future comparative genomic, transcriptomic and proteomic studies or novel protein/peptide discovery.

## Results

### The transcriptome of C. fleckeri

**Figure 1:**
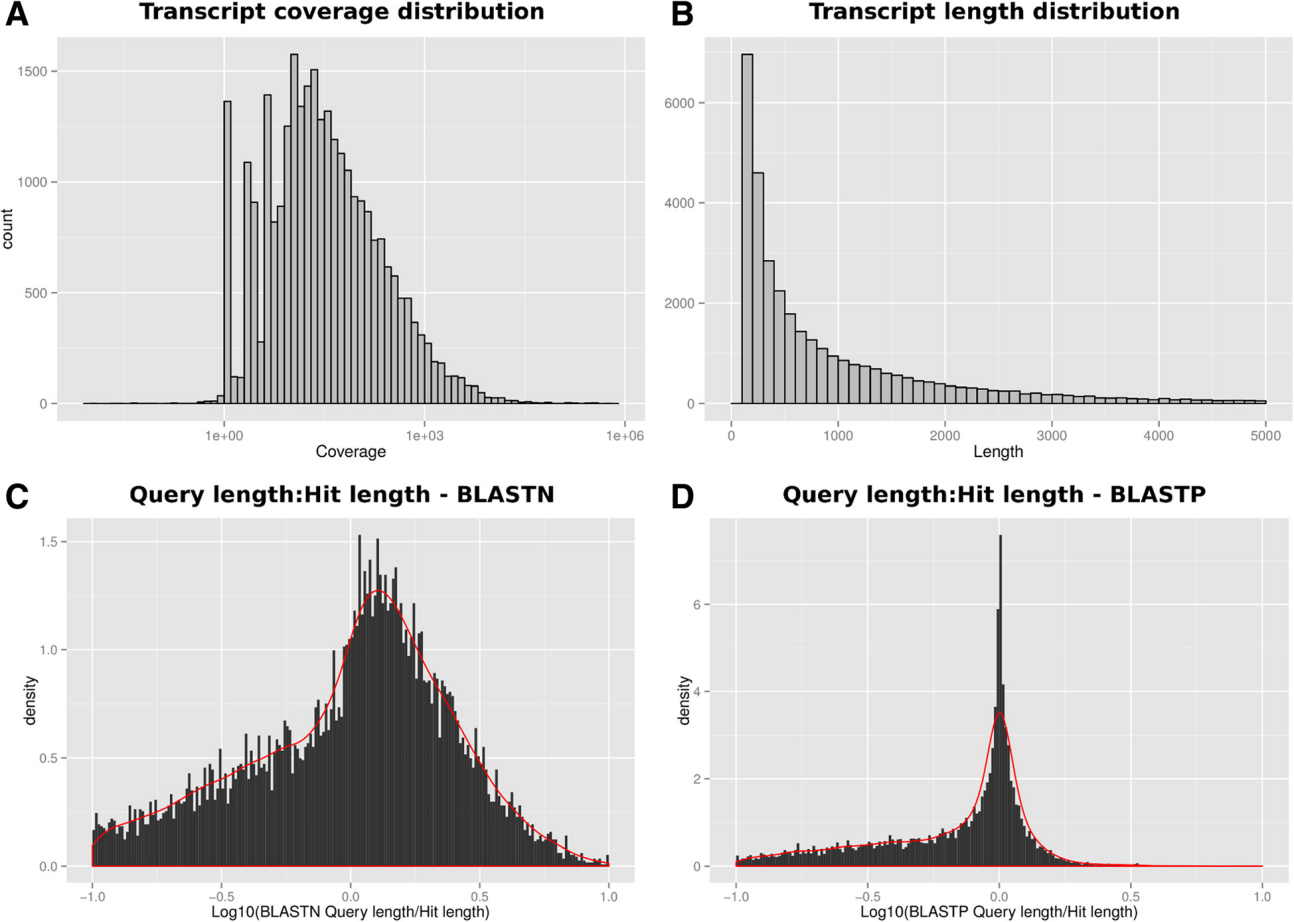
Summary of *C. fleckeri* assembly. **A**. The coverage of assembled transcripts after mapping of raw sequences back to the assembly using RSEM; **B**. The transcript length distribution; **C**. The distribution of the ratio of BLAST query length to BLAST hit length for transcripts when searched against a *H. magnipapillata* EST database using blastn. The EST database was obtained from Metazome (http://www.metazome.net/) and generated as part of the *H. magnipapillata* genome project; and **D**. Distribution of BLAST query length to hit length ratios for *C. fleckeri* predicted proteins searched against the SwissProt database using blastp.

Total RNA, purified from whole *C. fleckeri* tentacle tissue, was used to generate 43,150,858 paired reads using the Illumina platform. These reads were then *de novo* assembled, using Oases [9], into 34,438 transcripts that are summarized in Table 1. Approximately 56% (13,052,970) of the raw reads could be mapped back to the final assembly with a mean depth of coverage of 338.47 ± 6069.16 reads per sequence, although a proportion of assembled transcripts exhibited low read support (Figure 1A). Due to the limited number of cubozoan sequences available in protein databases, transcripts were searched against four databases using blastx — SwissProt, Cnidaria protein sequences from the GenBank non-redundant protein database and predicted protein sets from the *Hydra magnipapillata* and *Nematostella vectensis* genome projects. Approximately 40% of the sequences returned a high-scoring (e-value < = 10e-5) match to at least one of the databases (Table 1) and final annotations were assigned based on the match possessing the best bit score. A comparison of bit scores obtained from searches against protein databases from the model cnidarian organisms *H. magnipapillata* and *N. vectensis* suggested that, in general, *C. fleckeri* protein products were more similar to the former than the latter (Additional file 1: Figure S1). ESTScan, using a matrix constructed from annotated cnidarian sequences from the EMBL and GenBank databases, was used to identify 20,548 transcripts containing 20,562 predicted protein sequences that were used in MS/MS experiments. Of the remaining 13,890 transcripts not found to contain an open reading frame, only 1,587 had high scoring BLAST hits to proteins in one of the five databases used.

**Table 1:** **Summary of assembly and annotation of nucleotide sequence data from** ***Chironex fleckeri*** **tentacle tissue**

**Assembly**	
Raw reads (paired-end)	43,150,858
After clipping and QC	23,370,860
Contigs	34,438
Average length ± SD	1,056 ± 1359
Length min - max	100-26,403
% GC content	38.88
Raw reads mapped to contigs	13,052,970 (56%)
**ORFs**	
Transcripts with signficant BLAST hit (10e-5)	13736 (40%)
Containing an Open Reading frame	20,548 (60%)
With homologues in:	
*Nematostella vectensis*	12,143 (35%)
GenBank nr proteins (Cnidaria)	13,035 (38%)
*Hydra magnipapillata*	11,681 (34%)
SwissProt	11,123 (32%)
UniProt venom and toxins database	455 (1%)
**Matching CEGMA core eukaryotic proteins**	
% Full length (>90% cover)	77.02
% Partial (<90% cover)	80.65
**Interproscan**	
Returning Pfam terms	10,653 (31%)
Returning GO terms	7,208 (21%)
Total GO terms	17203
Biological Process	5,060
Cellular Component	2,745
Molecular Function	9,398
**Signal sequence and transmembrane domains**	
Predicted proteins with signal sequences	930 (3%)
Predicted proteins with > = 2 transmembrane domains	1,332 (4%)

The average length of assembled transcripts was 1056 ± 1359 bases (Figure 1B) with an N50 of 2123. To evaluate whether full-length sequences were present in the assembly, the length of transcripts with significant BLAST hits (e < = 10e-5) were compared to the length of their top BLAST hit. At the transcript level, sequences were compared to *H. magnipapillata* EST sequences using blastn and 63% of assembled transcripts were at least 80% the length of the BLAST hit (Figure 1C). At the protein level, the predicted protein set were compared to the SwissProt database using blastp and 54% of the proteins were at least 80% the length of the top hit (Figure 1D). Similarly, to estimate to what extent the actual transcriptome of *C. fleckeri* was covered by the assembled transcriptome, CEGMA [10] was used to identify core eukaryotic proteins. Approximately, 77% of the core CEGMA protein set was represented by full length transcripts (defined as > 90% cover) and 80% by partial transcripts (<= 90%) (Table 1). Predicted functions for proteins encoded by the assembled transcripts were assigned using InterProScan [11] and are summarized in Table 1, Additional files 2 and 3. Using SignalP [12] and TMHMM [13], approximately 2% (930) of the ESTScan predicted proteins contained signal sequences and 1,332 were predicted to contain two or more transmembrane domains.

### Toxin-like proteins identified in the transcriptome of C. fleckeri

**Figure 2:**
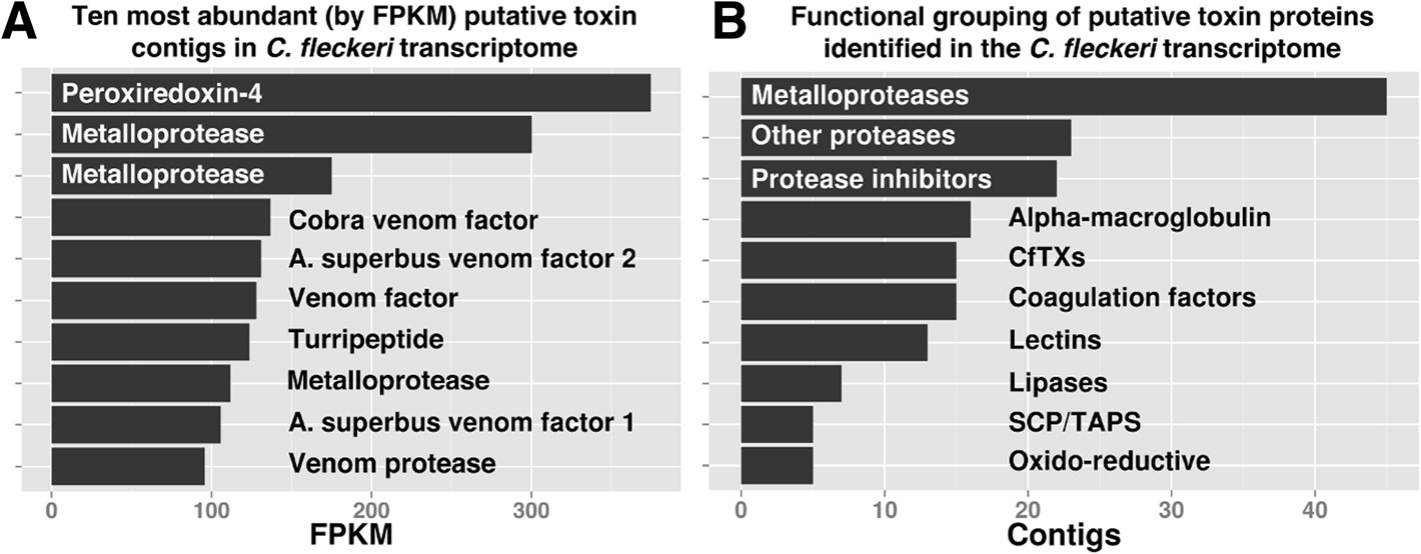
Potential toxin encoding transcripts identified in the transcriptome of *C. fleckeri*. **A**. The ten most abundant (by FPKM) transcripts encoding potential toxin proteins; **B**. Number of different transcripts in the assembly encoding potential toxin proteins from the ten toxin families identified in the transcriptome. In both **A**. and **B**. potential toxins were identified after screening against the SwissProt animal toxin database.

**Figure 3:**
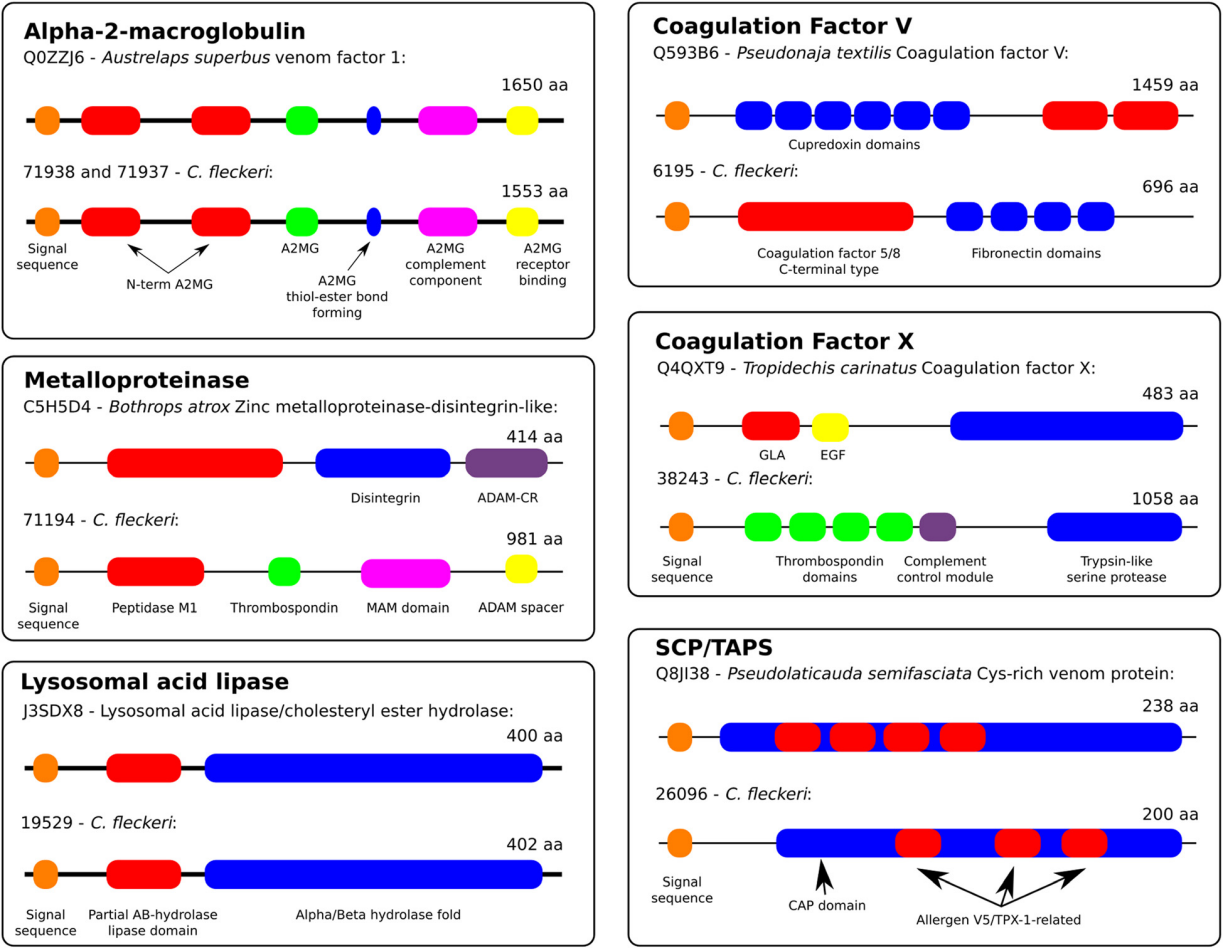
Domain structure of *C. fleckeri* transcripts encoding potential toxin proteins. Using InterProScan, the domain structure of potential *C. fleckeri* toxins were compared to their respective top scoring BLAST hit from the SwissProt animal toxin database. In three cases the domain structure of potential toxins was the same as those identified from the database but in the other cases different domain structures suggest functional divergence.

To identify potential *C. fleckeri* toxins, assembled transcripts were compared to the UniProt animal toxin database [14] using blastx. Four hundred and fifty-five transcripts (1%) provided high-scoring BLAST hits (bit score > 50). These potential toxins were further filtered: (1) those possessing a higher scoring BLAST hit (bit score) to a protein from a non-toxin protein family from the GenBank cnidarian database search (described above) were removed; and (2) proteins containing two or more transmembrane helices, as predicted by TMHMM, were removed. After filtering, 179 transcripts remained as putative *C. fleckeri* venom proteins (Additional file 4: Table S2), representing 10 venom protein families (Figures 2 and 3). Metalloproteinases, major constituents of spider venoms [15], were the most highly represented grouping with 45 different isoforms identified. Representatives of other toxin families included various proteases and protease inhibitors, lectins, lipases, CRISP venom proteins and two families of snake venom proteins — an alpha-macroglobulin-containing protein family with homologies to human complement protein C3 and toxins with homologies to human coagulation Factors X and V [16,17]. One hundred and eleven transcripts provided high-scoring BLAST matches to two other venom protein families; spider venom latrotoxins and snake venom calglandulins. However, in both cases these transcripts also provided high-scoring matches to non-venom proteins in GenBank, contained domains that were not specific to toxin proteins and did not contain a signal sequence; all of which suggest a role distinct from envenomation.

InterProScan was used to compare the domain structure of putative *C. fleckeri* venom proteins to representative examples identified during BLAST searches. Putative venom proteins from the alpha-2-macroglobulin, CRISP and lysosomal acid lipases families exhibited the same domain structure as found in characterized venom proteins (Figure 3). Metalloproteinases, and the two examples of coagulation factor-like proteins, exhibited different domain structures. For example, a *C. fleckeri* coagulation factor V homolog contained a C-terminal coagulation factor 5/8 domain at the N-terminus of the protein with four fibronectin domains whereas the snake venom toxin contained six cupredoxin domains followed by the C-terminal coagulation 5/8 domain (Figure 3). Abundance of putative toxin proteins was assessed using the FPKM and a peroxiredoxin isoform (75536) and an astacin-like metalloproteinase isoform (71187) were the two most abundant toxin transcripts. Also abundant were other astacin-like metalloproteinases, three alpha-macroglobulin-containing snake venom proteins and four snake venom factors (Figure 2B).

Molecular studies of jellyfish venom have gradually revealed the existence of a novel, cnidarian toxin family [18-23]. In *C. fleckeri* characterized members of this family include CfTX-1, −2, −A and -B, all of which are abundantly present in the venom of *C. fleckeri* [6]. In this work, 15 CfTX isoforms were identified, including CfTX-A, −B, −1 and −2. Three novel CfTX proteins were identified as full length transcripts, a protein with homologies to TX-1 from the scyphozoan jellyfish *Aurelia aurita* (id 32640), a protein similar to CfTX-A (id 37616) and a truncated protein similar to CfTX-2 (id 32230). Partial sequences for nine other toxin proteins were identified in the assembly, including two CfTX-1-like toxins, five CfTX-2-like toxins and two toxins similar to CqTX-A from the box jellyfish *Chironex yamaguchii* (Table 1 and Suppl. Data 2). A truncated form of CfTX-B has been reported, CfTX-Bt [2], and although this transcript was not identified in the assembly a similarly truncated form of CfTX-1 was identified (10226). The latter contained a signal sequence and a domain similar to the N-terminal domain of CfTX-1.

### Proteomic analysis of C. fleckeri venom proteins

**Figure 4:**
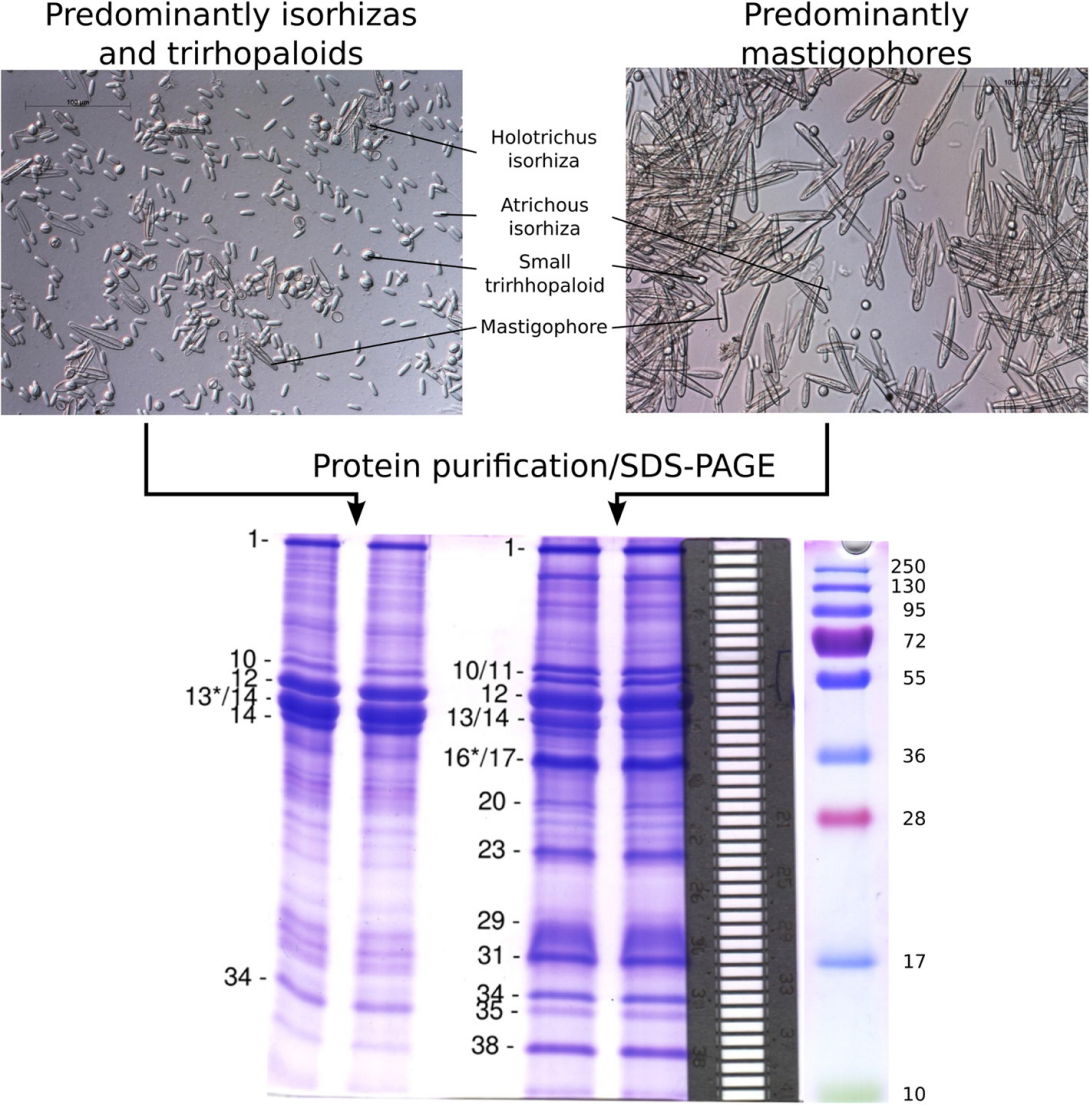
Light microscopy of nematocysts used in proteomic analysis and SDS-PAGE gels of venom proteins. Two nematocyst preparations were analyzed; a preparation containing predominantly mastigophores (right) and a preparation enriched in isorhizas and trirhopaloids (left). Different morphological types are indicated (magnification 200x). Extracts of the nematocyst preparations were fractionated using SDS-PAGE (bottom) and proteins identified using tandem mass spectrometry. Forty-one gel slices were excised from each lane, as indicated by the aligned metal grid (right). Selected gel slices corresponding to major protein bands are indicated on the gel. Where a protein band was divided between two gel slices, an asterisk denotes the gel slice containing the majority of that protein.

**Figure 5:**
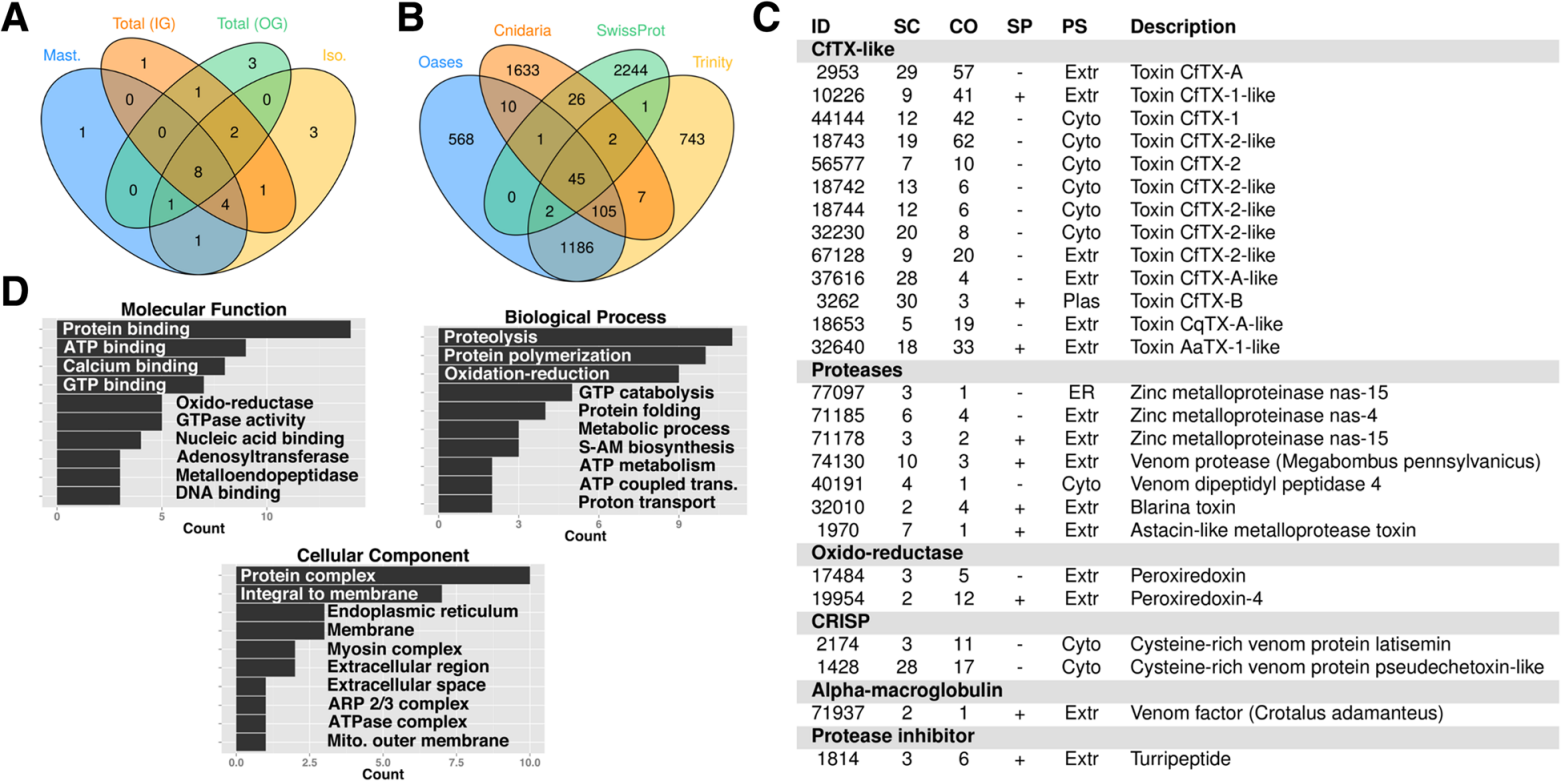
MS/MS analysis of *C. fleckeri* venom. **A**. Venn diagram showing the numbers of potential toxin proteins identified in each MS/MS experiment. Abbreviations used, Mast. — nematocyst sample containing predominantly mastigophores; Iso. — nematocyst sample containing predominantly isorhizas and trirhopaloids; Total (IG) — total nematocyst sample fractionated using SDS-PAGE; and Total (OG) — total nematocyst sample fractionated using peptide OFFGEL electrophoresis; **B**. Venn diagram showing overlap in significant peptide identifications in three additional databases searches using MS/MS data. The databases depicted are 1.) Oases — predicted protein dataset from Oases assembly; 2.) Trinity — predicted dataset Trinity assembly; 3.) Cnidaria — all cnidarian proteins from the GenBank non-redundant protein database; and 4.) SwissProt — the Uniprot SwissProt database; **C**. Proteins identified in the venom using MS/MS that had been previously identified as potential toxins during the analysis of the transcriptome; **D**. GO terms associated with proteins identified in the venom of *C. fleckeri* using MS/MS.

To confirm which putative toxin proteins were present in *C. fleckeri* venom, MS/MS was used to identify proteins from nematocyst-derived protein preparations. Total protein from two nematocyst preparations, each containing morphologically distinct classes of nematocyst, were analyzed using MS/MS; (1) a preparation containing predominantly mastigophores, nematocysts believed to hold the lethal venom components [24]; and (2) a preparation enriched in isorhizas, thought to have a non-penetrative role in entangling prey [24], and trirhopaloids, penetrative nematocysts with an undetermined role in envenomation. Nematocyst preparations were fractionated using SDS-PAGE and proteins identified using MS/MS and searches against the predicted protein set from the transcriptome (Figure 4). In addition, existing MS/MS data sets, generated during our previous studies of *C. fleckeri* venom [6], were reanalyzed using the new transcriptomic data. These data sets included duplicate in-gel digests of SDS-PAGE-fractionated venom proteins from total nematocyst preparations and duplicate peptide OFFGEL™ electrophoresis experiments of the same samples (Figure 5A).

More than 507,945 spectra from in-gel digests and OFFGEL™ experiments were used in X! Tandem searches of the predicted proteins. Across all experiments, 263 proteins were identified and these were categorized into eight functional groupings (Additional file 5: Table S3). Proteins identified included 26 of the putative toxins identified in transcriptomic analysis (Additional file 5: Table S3). In addition to toxins, structural proteins were highly represented (57 proteins) as well as 84 proteins from diverse functional families that were grouped into the ’Miscellaneous’ category. Twenty-three uncharacterized proteins were identified, ten of which were identified by five or more unique peptides and/or were identified in at least two of the four experiments (Additional file 5: Table S3). The relatively high spectral counts and reproducible identification of these proteins suggests that they are jellyfish-specific proteins and thus are potential novel constituents, toxin or otherwise, of the venom proteome. To increase the completeness of the proteomic analysis and to account for deficiencies in the Oasis assembly, we conducted X! Tandem searches against three other protein databases; (1) ESTScan predictions from a transcriptome assembled from the same reads using Trinity [25]; (2) all Cnidaria proteins from the GenBank non-redundant protein database; and (3) the complete SwissProt database. Figure 5B shows the global comparison of peptide identifications from the four databases. Each database search added a set of unique protein identifications and, using sequence based comparisons (blastp evalue < = 0.00005 and hsp percent identity > =90%) 168 additional proteins were added to the proteome; 25 from Trinity ORFs, 31 from the Cnidaria protein database and 112 from the SwissProt database, most of which were common contaminants of proteomic experiments (Figure 5B); Additional file 6: Table S4). Apart from one identification of CfTX-1, corresponding to the full length sequence of the partial sequence assembled using Oases, no additional toxin proteins were identified.

‘Molecular Function’ and ‘Biological Process’ GO terms highly represented among the identified proteins included, “oxidation-reduction”, “protein binding”, “proteolysis” and “ATP binding” (Figure 5D). Based on SignalP and TMHMM analysis, 46 proteins were inferred to contain a classical secretory signal peptide, with the ‘Toxins’ and ‘Uncharacterized’ functional groupings containing the greatest proportion of secreted proteins (36% and 39% respectively). Only eight proteins were predicted to contain more than two trans-membrane domains. Cellular location was further analyzed using PSort [26]; approximately 43% of the identified proteins were predicted to be cytoplasmic and 19% extracellular in origin, with the remainder originating from the mitochondria, cytoskeleton, nucleus and endoplasmic reticulum. The ‘Toxins’ functional grouping had the highest proportion of extracellular proteins (54%) (Additional file 5: Table S3).

### Toxin proteins identified in C. fleckeri venom using tandem mass spectrometry

Twenty-six putative toxin proteins were identified in the venom of *C. fleckeri* using MS/MS (Figure 5C) and all had been previously identified as putative toxins during analysis of the transcriptome. The majority of these toxins were members of the CfTX toxin family with 13 of the 15 isoforms identified in the transcriptome also identified using MS/MS. Five CfTX proteins (CfTX-1, −2, −A, −B and -Bt) have been previously characterized and all but one, CfTX-Bt, were identified here. CfTX-Bt, which has high sequence homology to CfTX-B but is distinguished by C-terminal truncation was not identified, most likely due to its homology to CfTX-B, which was identified. Of the nine uncharacterized CfTX proteins identified in the MS/MS data, seven showed greatest homology (i.e. highest BLAST score) to the characterized *C. fleckeri* toxin proteins and two showed the greatest similarity to characterized toxins of the same family; CaTX-A from the cubozoan *Chironex yamaguchii* and TX-1 from the scyphozoan *A. aurita*. The 13 other putative toxins identified included seven proteases, four of which were metalloproteinases, an alpha-macroglobulin domain containing protein, two peroxiredoxin toxins, two CRISP proteins and a turripeptide-like protease inhibitor (Figure 5C). Although not annotated as toxins, a further 12 proteases, including carboxypeptidases, endothelin-converting enzymes and two collagenases were identified (Additional file 5: Table S3). In total four different nematocyst preparations were analyzed, and toxins were reproducibly identified with 79% of the toxins detected in at least two of the five experiments (Figure 5A). The toxin content of the two different nematocyst preparations was similar with 15 toxin proteins identified in the mastigophore preparation and 20 in the isorhiza and trirhopaloid nematocysts.

### Comparison of the C. fleckeri venom proteome to those from other cnidarians

**Figure 6:**
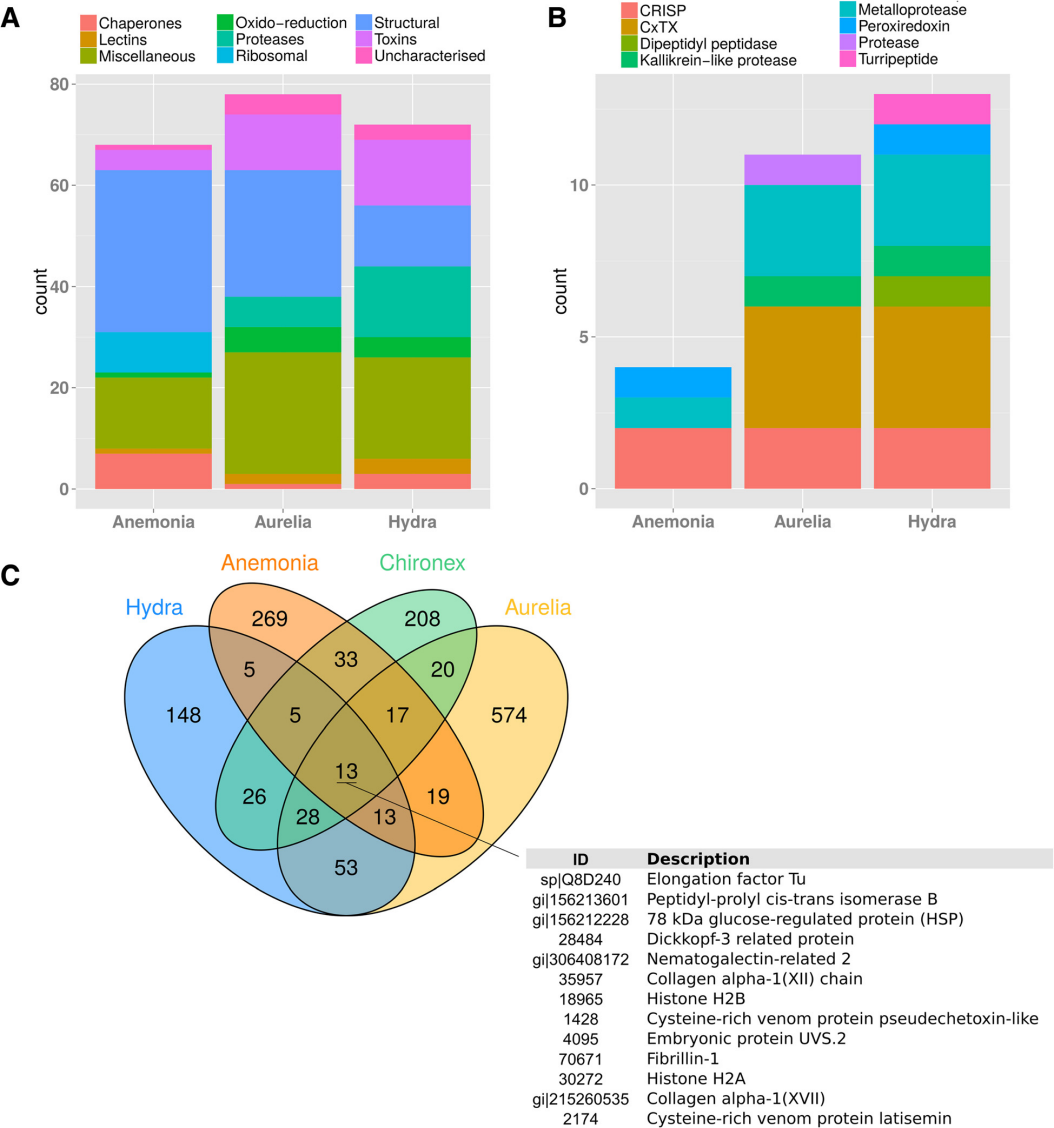
Comparison of cnidarian venom proteomes. **A**. Proteins from the *C. fleckeri* proteome with corresponding proteins in *H. magnipapillata*, *A. aurita* and *A. viridis* by functional category; **B**. Potential toxins from *C. fleckeri* with corresponding proteins in the same species; **C**. Venn diagram depicting the overlap in proteins identified in the proteomes of four cnidarian species.

**Figure 7:**
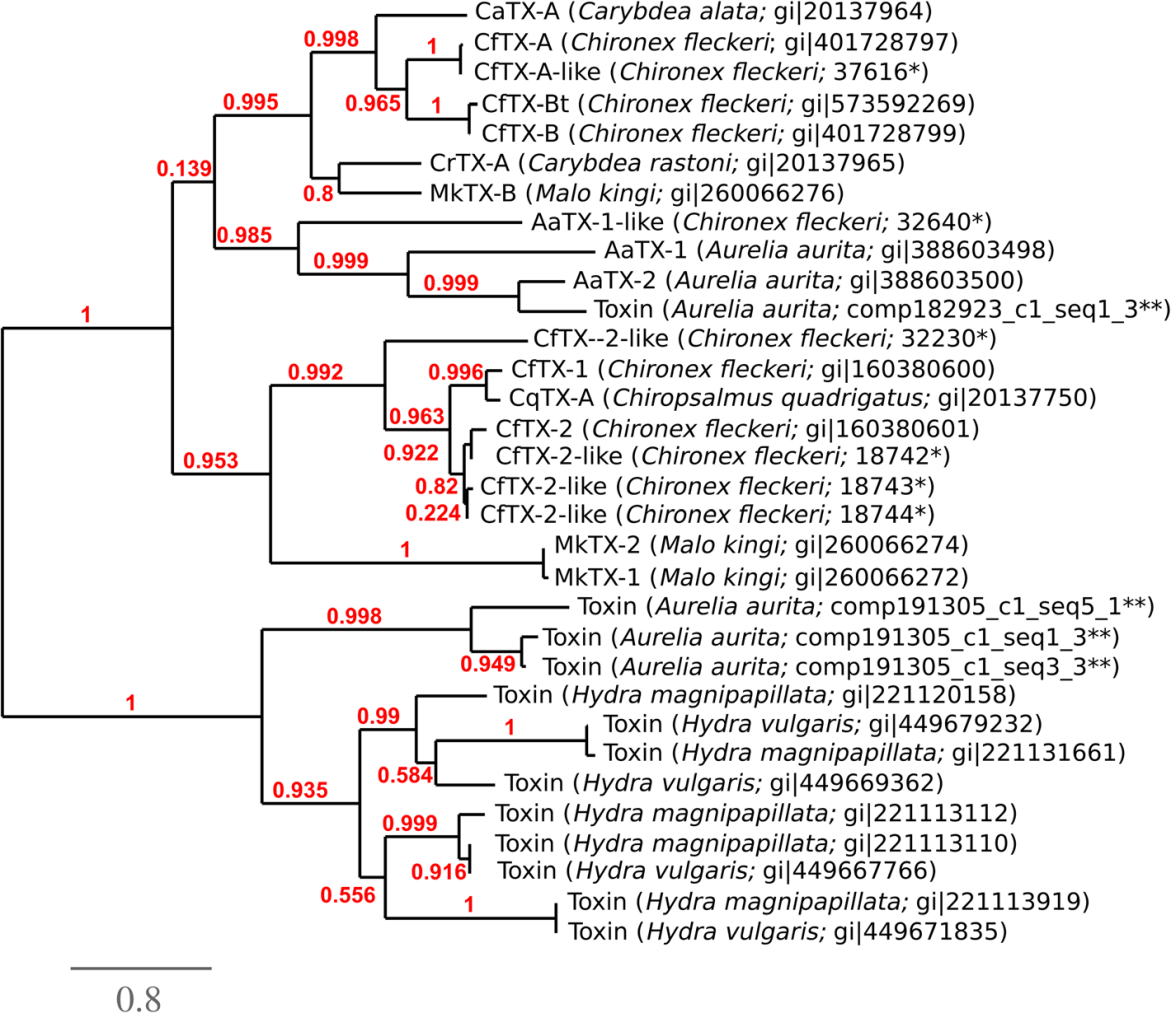
Phylogenetic tree of characterized CfTX toxin proteins. Phylogenetic tree depicting the grouping of CfTX-like proteins in cnidaria. Proteins names and accessions are shown. Proteins identified in this study are depicted with an asterisk and those from [27] with a double asterisk. The tree was produced using MUSCLE and PhyML for tree building and the aLRT statistical test [55] was used for branch support.

The venom proteome of three other cnidarians were recently characterized: the anthozoan sea anenome *Anemonia viridis*, the schyphozoan jellyfish *Aurelia aurita* and the hydrozoan Hydra magnipapillata [27]. To place the cnidarian venom proteome described here in this context we compared the venom proteome of all four species. Using blastp, proteins from these organisms with significant (e-value < 10e^−5^) homology to *C. fleckeri* venom proteins were identified and functionally categorized. *C. fleckeri* proteins most commonly found in the venom proteome of the other cnidarians were predominantly structural or were classed as miscellaneous (Figure 6A). Overall, *A. aurita* had the most shared proteins, followed by *H. magnipapillata* and *A. viridis* (Figure 6A). To compare the toxin repertoire of the four cnidarians the same analysis was conducted for putative toxins identified in the *C. fleckeri* proteome. Both *H. magnipapillata* and *A. aurita* possessed similar classes of putative toxins as identified in *C. fleckeri*. This included CfTX-like proteins that were well represented in all three organisms (Figure 6B). Conversely, few toxin proteins were identified in both *A. viridis* and *C. fleckeri* and no CfTX-like proteins were identified in the former (Figure 6B). As previously reported for *H. magnipapillata*, *A. aurita* and *A. viridis* [27], very few proteins (13) were identified in all four species (Figure 6C). A phylogenetic tree of CfTX and CfTX-like proteins showed three main groupings of the toxin proteins, represented by CfTX-A and -B, CfTX-1 and −2, and a third group represented predominantly by toxins from *Hydra* species (Figure 7).

## Discussion

The primary aim of this study was to identify protein toxins responsible for the severe effects of *C. fleckeri* stings in humans. It has been previously noted that the great diversity in animal toxin function has evolved from a limited number of protein families [28], thus we used sequence homology to known animal toxins, from the UniProt animal toxin database, as a strategy for the identification of potential toxins from the transcriptome of *C. fleckeri*. We then confirmed the existence of a subset of these proteins in the venom proteome using MS/MS. In the absence of a reference genome, we generated a *de novo* assembly using a methodology designed to maximize reference coverage while minimizing redundancy and chimera rate [29]. CEGMA analysis suggested a reasonable coverage of the transcriptome was achieved using this approach, although a proportion of sequences (37% of transcripts and 46% of predicted proteins) (Figure 1C and D) did not represent at least 80% of their highest scoring blast hit, suggesting that further sequencing is required to fully characterize the transcriptome. Despite this caveat, the final assembly provided a sufficient quantity of full- and partial-length transcripts for the identification of major toxin families present in the transcriptome and for the generation of a set of predicted proteins suitable for use in proteomics searches. These searches revealed the presence of proteins with homology to at least ten known toxin families including metalloproteinases, protease inhibitors, alpha-macroglobulins and CfTX proteins. Although screening for known toxin families will not reveal the presence of novel jellyfish-specific toxins, identification of metalloproteinases, CfTX proteins and protease inhibitors in the proteomic analysis suggests that some of the protein toxin families present in *C. fleckeri* venom have been identified using this strategy.

The *de novo* assembly of short read Illumina data is still a challenge. In this work, 44% of the short reads did not map back to the assembly and, despite a mean coverage of 332 reads per transcript, a subset of transcripts had poor read support (Figure 1B), suggesting that that a complete description of the assembly has not been achieved. This not only reflects the inherent challenges in the *de novo* assembly of transcriptomes from short reads, including variations in transcript expression, sequence biases from next generation sequencing, alternative splicing and overlapping genes, but also the quality control strategies employed during assembly, including the methodology adopted from [29] and the clustering of transcripts after assembly. To identify and remedy potential gaps in our assembly we conducted proteomic searches against multiple protein databases, and these searches provided 168 additional protein identifications over the searches against the Oases assembly alone. These included nematocyst proteins, such as nematogalectins, dickkopf-related proteins and endothelin-converting enzymes (Additional file 6: Table S4), previously shown to be abundant in venom preparations [6], suggesting that this approach provided better proteome coverage than would have been achieved using the Oases assembly alone.

Our comparison of the *C. fleckeri* venom proteome with those of *H. magnipapillata* and *A. aurita* shows that they have a functionally similar toxin repertoire, dominated by cytolysins and proteases. This is in contrast to the venom of the anthozoan *A. viridis*, which is rich in low molecular weight neurotoxins that are not typically found in medusozoans [27]. The proteomic characterization the venom of another scyphozoan, *Stomolophus meleagris* [30], was broadly similar to the three medusozoans compared in this study. In this organism serine proteases and phospholipases were the most abundant classes of toxins followed by proteins containing a ShK toxin domain and a number of other haemolysins. Although we did not identify any phospholipases or ShK-domain containing proteins in the proteome of *C. fleckeri*, several types of both proteins were identified in its transcriptome. In both organisms metalloproteinases, lectins and serine protease inhibitors where identified in the venom proteome and are potential toxin proteins.

In *C. fleckeri*, the CfTX proteins have undergone an expansion with fifteen examples identified in the transcriptome and thirteen in the venom proteome. Members of this toxin family are potently haemolytic and cause pain, inflammation, dermonecrosis, cardiovascular collapse and death in experimental animals [2,5,18-21,23], suggesting that these toxins are responsible for many of the symptoms of box jellyfish envenomation. While these toxins are highly abundant in cubozoan venoms [19-22], their distribution among other medusozoans appears to be less uniform. For example, CfTX-like toxins have been identified in the venoms of *A. aurita*, *C. capillata* and *H. magnipapillata* [27,31] but not in the venoms of *S. meleagris* [30] or the hydrozoan *Olindias sambaquiensis* [32]. Their abundance in *C. fleckeri*, and related cubozoans [19-22] and the relatively few reports from Class Scyphozoa could explain why cubozoan stings are generally more severe than those of scyphozoan species. Alternatively, phylogenetic analysis of the CfTXs shows that the toxins fall into two main groups: a clade of mainly hydrozoan toxins and a clade of predominantly cubozoan toxins that branches into two smaller subclades (Figure 7). Examples from the scyphozoan *A. aurita* are present in the *Hydra* clade and one of the Cubozoa subclades. This pattern suggests that the toxins have undergone functional and structural diversification during evolution that could be associated with differences in the toxicity of various jellyfish species [2].

Identifying potential toxins from sequence homology is complicated by the evolutionary history of some toxins that evolved from proteins with roles unrelated to envenoming. This is particularly acute when, as in this study, RNA from the entire tentacle is used to generate an assembly that includes transcripts from tissue not specifically involved in envenomation. Even proteins identified in the venom proteome could be involved in biological processes unrelated to envenomation. For example, in *Hydractinia echinata*, on the basis of phylogenetic and *in situ* hybridization experiments, astacin metalloproteinases have been implicated in development [33], although a function in digestion has also been proposed [34]. Similarly, it has been proposed that peroxiredoxins in snake venom eliminate peroxides generated during metabolism and thus maintain redox homeostasis [35]. Therefore the two peroxiredoxins identified in *C. fleckeri* could play a similar role, as is the case in the scyphozoan jellyfish *Cyanea capillata* [36]. Conversely, proteins not designated as potential toxins at the transcript level, were identified in the proteome and could be toxins. This included thirteen additional proteases and a number of uncharacterized proteins with very high spectral support (Additional file 5: Table S3). As members of the venom proteome, all of these proteins are presumably transferred during a sting and could thus contribute to the symptoms of envenoming. However, an exact determination of their biological role in inducing the symptoms of envenomation now awaits further experimental validation.

Mastigophores are thought to contain the lethal protein components of *C. fleckeri* venom [24], while isorhizas are considered non-penetrative with adhesive or entanglement functions and could thus possess a different toxin profile. In this work, however, there was little difference in the toxin content of the two nematocyst preparations. This heterogeneity could be a result of incomplete segregation of the different nematocyst classes during sample preparation (see Figure 4 for example), that the penetrative trirhopaloid nematocysts contain similar toxins as the mastigophores or that the toxin proteins are present in all forms of nematocysts, regardless of penetrative ability. A more stringent approach, for example a quantitative proteomics study, would be required to accurately determine the type and relative abundance of toxins that are present in each nematocyst class.

Other protein classes highly represented in the proteome of *C. fleckeri* included structural, ribosomal and oxido-reductive proteins as well as proteins of diverse function that were grouped into a ‘miscellaneous’ category. The identification of structural proteins may reflect the way that the venom was purified from the intact nematocysts. DTT, a strong reducing agent, was used to partially disintegrate the nematocyst capsule and cause venom release, so it is likely that a proportion of capsular components and other structural proteins were also solubilized during this process. On the other hand, the identification of ribosomal, oxido-reductive and the ‘miscellaneous’ proteins are likely a result of the manner in which nematocysts are formed. A nematocyst is formed within a large post-Golgi vesicle [37] and some proteins are likely to be incorporated into the venom as a consequence of their presence during nematocyst formation. The relative abundance of these proteins, along with the structural proteins that are liberated from the nematocyst capsule during venom extraction, can mask the presence of lower abundance proteins that may be active at nanomolar concentrations (for example some snake venom serine proteases [38]). Hence, it is possible that low-abundance toxins, that could contribute to the symptoms of envenoming were not identified in the MS/MS experiments. This is supported by single peptide identifications (not reported here), which included known nematocyst proteins and potential toxins such as nematoblast-specific protein nb035-sv3 and lectoxin. These single peptide identifications suggest that the reliable identification of lower abundance proteins will expand the catalogue of known *C. fleckeri* toxins. Experiments are currently underway to identify these low-abundance toxins by improving methods of venom purification to decrease the proportion of structural proteins in the purified venom and by improving the fractionation of toxins before tandem MS/MS.

## Conclusion

*C. fleckeri* produces one of the most potent venoms known to man but its protein composition is poorly understood compared to those of other well characterized animals, such as snakes, scorpions and spiders. This work presents the first transcriptome from a cubozoan jellyfish from which the venom proteome has been refined. This work provides the basis for a range of studies aimed at 1.) improving medical responses to envenoming; 2.) exploring the potential of these toxins as a source of novel bioactive compounds; and 3.) conducting comparative proteomic and transcriptomic studies of other cubozoan jellyfish of medical importance, such as the Irukandji.

## Methods

### Jellyfish collection

Jellyfish were captured at Balgal Beach and Weipa (Queensland, Australia) by the Queensland Surf Life Saving Association and Christopher Mooney (James Cook University). No specific permits were required for the described field studies. No specific permissions were required as the animals collected are not protected and were collected from marine environments that are not protected or privately owned. *C. fleckeri* is not an endangered or protected species.

### RNA isolation and Illumina sequencing

Tentacles were excised and immediately preserved in RNAlater (Life Technologies). Total RNA was isolated from the *C. fleckeri* tentacles of a single specimen using a handheld rotor-stator homogenizer (QIAgen) and TRIzol (Life Technologies), according to the manufacturer’s instructions. The RNA was DNAse-treated, purified using a Nucleospin RNA II kit (Macherey-Nagel) and eluted in nuclease-free water. RNA purity, concentration and integrity were evaluated using a NanoDrop 2000 UV–vis spectrophotometer and an Agilent 2100 Bioanalyzer with a RNA 6000 Pico kit. Purified RNA (5 μg; RIN 9.1) was submitted to the Ramaciotti Centre for Genomics (Sydney, Australia) for RNA-seq library construction and 100 bp paired-end sequencing on an Illumina HiSeq2000 sequencer. RNA sequence data have been submitted to the Sequence Read Archive (National Center for Biotechnology Information, U.S. National Library of Medicine, Bethesda, MD) under accession number PRJNA276493.

### Transcriptome assembly

Adapter sequences and low quality (Phred score < 32) bases were clipped from 100 bp paired-end sequences using Trimmomatic [39] and reads less than 75 bases were discarded. Initial quality assessment was performed using FastQC (http://www.bioinformatics.babraham.ac.uk/projects/fastqc/). To ensure a high quality assembly and to reduce the number of chimeric contigs, multiple assemblies using different k-mer values were generated as described [29]. Briefly, reads remaining after quality control were used to construct de Bruijn-graphs with k-mer values of 21, 31, 41, 51 and 61 using Oases v.0.2.09 [9]. Transcripts less than 0.3 in length of the longest transcripts in the same locus were then filtered and transcripts from k = 21, 31, 41, 51 were accepted if there was either 1 or 3 transcripts per locus. No limit was applied for transcripts per locus at k = 61. Redundancy was removed from the final collection of transcripts using CD-EST [40] with the sequence identity cut-off set to 0.98 (−c 0.98 -n 10 -r 1). For a comparison to the Oases assembly another assembly was generated using Trinity [25]. This assembly used the same raw reads as the Oases assembly and was constructed using the default parameters. To obtain relative abundance estimates the program RSEM [41] was used with the non-redundant sequences. Using RSEM, raw reads were mapped to a reference database generated from the assembled transcripts and maximum likelihood abundance estimates were obtained using the Expectation-Maximization algorithm as a statistical model. Final abundance estimates were calculated as Fragments Per Kilobase of exon per Million fragments mapped (FPKM).

### Annotation of transcripts

Oases assembled transcripts were then compared (using tBLASTx and BLASTx; e-value threshold of < 10e-5) to sequences available in public databases. Sequences were compared to (1) Swiss-Prot (as of the 1st of Oct. 2013), (2) Cnidaria protein sequences from the GenBank non-redundant protein database; (3) and the complete genomes and transcriptomic data sets of *Hydra magnipapillata* [42] and *Nematostella vectensis* [43] from Metazome (http://www.metazome.net/). The bit scores from the highest scoring match from each database were then compared and a final annotation assigned based on the match with the highest bit score. Transcripts encoding potential toxin proteins were identified using BLASTx against the UniProt animal toxin database [14] (http://www.uniprot.org/program/Toxins) and those with a high-scoring match (bit score > 50) that did not have a better scoring match from the GenBank cnidarian protein database to a non-toxin protein family were designated as a potential toxin. For protein sequence prediction, the program ESTScan [44] was used to distinguish coding from non-coding sequence. ESTScan uses Hidden Markov Models to detect hexanucleotide biases in coding sequence, so prior to analysis, established methods were used to construct a scoring matrix from 26,484 annotated Cnidarian mRNA sequences from the RefSeq (http://www.ncbi.nlm.nih.gov/refseq/) and EBI (http://www.ebi.ac.uk/ena/) databases [44]. Sequences not providing a predicted coding sequence using ESTScan but which provided a BLAST hit with an e-value less than 10e-5 were translated in the appropriate reading frame and added to the predicted protein set. The final set of protein sequences were analyzed with InterProScan [11] using the default search parameters. Based on their homology to conserved domains and protein families, proteins were assigned parental (i.e., level 2) gene ontology (GO) terms (i.e., ‘biological process’, ‘cellular component’ and ‘molecular function’) (http://www.geneontology.org/) [45]. Inferred proteins with homologues in other organisms were mapped to conserved biological pathways utilizing the Kyoto Encyclopedia of Genes and Genomes (KEGG) Orthology-Based Annotation System v.2 (= KOBAS2) [46]. Signal peptides were predicted using the program SignalP 4.0 [12], employing the neural network and hidden Markov models and transmembrane domains were inferred using the program TMHMM [13] (http://www.cbs.dtu.dk/services/TMHMM/).

### Nematocyst isolation

Nematocysts were isolated from excised tentacles as previously described [47], except the nematocysts were not lyophilised. Nematocysts were purified from tentacle debris by centrifugation (300 x g, 1 h, 4°C) in a discontinuous gradient of Percoll (Sigma) comprising three layers of 100%, 90% and 30% Percoll, with 35 g/L NaCl as the diluent. Nematocysts were recovered from the 30-90% boundary, washed repeatedly in 35 g/L NaCl and resuspended in 35 g/L NaCl. The integrity of the undischarged nematocysts was verified using an Axioskop2 mot plus light microscope (Zeiss). A subsample of nematocysts was allowed to settle (2-3 h, 4°C). The supernatant containing lower density nematocysts was transferred to another tube and re-examined microscopically.

### Electrophoresis and in-gel digestion

Venom was prepared from Percoll-cleaned nematocysts as previously described [6]. Briefly, nematocysts were washed in low salt buffer, resuspended 1:6 (wet w/v) in reducing SDS-sample buffer [48] containing diothiothreitol (DTT) and incubated at room temperature until ≥90% nematocyst discharge was observed microscopically. Capsular debris was removed by centrifugation (16 k x g, 10 min, 4°C). Supernatants were transferred to clean tubes and heated (95°C, 5 min). Duplicate samples (8 μl) were each applied to a single well of a 15% SDS-PAGE gel and electrophoresis performed according to Laemmli [48]. Proteins were stained with Coomassie Brilliant Blue R-250 staining solution (Bio-Rad) and each sample lane was cut into 41 gel slices using a 1.5 mm x 5 mm GridCutter (Gel Company). The gel slices were then destained twice in 200 μl of 50% acetonitrile, 200 mM ammonium bicarbonate for 45 min at 37°C, desiccated using a vacuum centrifuge and then resuspended in 20 mM DTT, 25 mM ammonium bicarbonate and reduced for 1 h at 65°C. DTT was then removed, and the samples were alkylated in 50 mM iodoacetamide and 25 mM ammonium bicarbonate at 37°C in darkness for 40 min. Gel slices were washed three times for 45 min in 25 mM ammonium bicarbonate and then desiccated. Individual dried slices were then allowed to swell in 20 μl of 40 mM ammonium bicarbonate, 10% acetonitrile containing 20 μg/ml trypsin (Sigma) for 1 h at room temperature. An additional 50 μl of the same solution was added and the samples were incubated overnight at 37°C. The supernatants were removed from the gel slices, and residual peptides were washed from the slices by incubating them three times in 50 μl of 0.1% formic acid for 45 min at 37°C. The original supernatant and washes were combined and reduced to 10 μl in a vacuum centrifuge before mass spectral analysis.

### Protein identification using LC-MS/MS

Tryptic fragments from in-gel digests were separated chromatographically by a Eksigent cHiPLCTM-nanoflex system using a 15 cm long chromXP C18-CL column (particle size 3 μm, 120 Å, 200 μm x 6 mm) and a linear gradient of 0-95% solvent B for 72 min. A pre-concentration step (10 min) was performed employing a chromxp trap (C18-CL, 3 μm, 120 Å, 200 μm x 6 mm) before commencement of the gradient. A flow rate of 500 nl/min was used for all experiments. The mobile phase consisted of solvent A (0.1% formic acid [aq]) and solvent B (100 acetonitrile/0.1% formic acid [aq]). Eluates from the RP-HPLC column were directly introduced into the NanoSpray II ionisation source of a TripleTOF 5600 MS/MS System (AB Sciex) operated in positive ion electrospray mode. All analyses were performed using Information Dependant Acquisition. Analyst 2.0 (Applied Biosystems) was used for data analysis. Briefly, the acquisition protocol consisted of the use of an Enhanced Mass Spectrum scan with 10 seconds exclusion time and 50 mDa mass tolerance. A cycle time of 2800 ms was used to acquire full scan TOFMS data over the mass range 320–2000 m/z and product ion scans over the mass range of 100–2000 m/z for up to 25 of the most abundant ions with a relative intensity above 100 and a charge state of +2 − +4. Full product ion spectra for each of the selected precursors were then used for subsequent database searches. Proteomic datasets are deposited in ProteomeXchange with the accession number PXD002068.

### Bioinformatic analyses of proteomic sequence data

In addition to the in-gel digests performed as part of this study existing MS/MS data sets, generated during our previous studies of *C. fleckeri* venom [6], were reanalyzed using the new transcriptomic data. These data sets included duplicate in-gel digests of SDS-PAGE-fractionated venom proteins from total nematocyst preparations and duplicate peptide OFFGEL^™^ electrophoresis experiments of the same samples. For the primary analysis, spectra from all datasets were used to search the ESTScan-predicted protein coding sequences from the Oases transcriptomic assembly (20,548 proteins). In addition, all in-gel datasets were searched against three other databases to assess the completeness of the primary analysis; (1) ESTScan predicted proteins from a second assembly constructed using Trinity (30,245 proteins); (2) a database of all Cnidarian proteins in the GenBank non-redundant protein database (as of the 1st of Oct. 2013; 95,394 proteins); and (3) the SwissProt database (as of the 1st of Oct. 2013; 546,000 proteins). All searches were conducted using X! Tandem v.2013.09.01.1 [49], employing the following search parameters: enzyme = trypsin; precursor ion mass tolerance = ± 0.1 Da; fragment ion tolerance = ±0.1 Da; fixed modifications = carbamidomethylation; variable modifications = methionine oxidation; number of missed cleavages allowed = 2; and allowed charge states = +2 − +4. In the primary analysis, against the Oases predicted protein set, MS/MS data from each band of the in-gel digests were searched individually (Additional file 7: Table S5). For OFFGEL samples, data from each fraction was combined for X! Tandem searches and the Trans Proteomic Pipeline [50] was used to validate peptide and protein identifications using PeptideProphet [51] and ProteinProphet [52] (Additional file 8: Table S6). False discovery analysis for OFFGEL samples, calculated as less than 1% for reported proteins, was conducted using Mayu [53] and searches against a database comprising the predicted protein set from the Oases assembly and the reverse of each sequence (Additional file 9). Proteins containing similar peptides but which could not be differentiated based on MS/MS analysis were grouped to satisfy the principles of parsimony. For in-gel digests and OFFGEL experiments, proteins were reported only if two significant peptides (p < 0.05) were attributed to the protein, at least one of which was unique to that protein. The phylogenetic tree was produced using MUSCLE for multiple alignment, Gblocks for automatic alignment curation, PhyML for tree building and TreeDyn for tree drawing using the tree-generation pipeline at Phylogeny.fr website [54]. The aLRT statistical test [55] was used for branch support.

## Additional files

Additional file 1:
**Comparison of** ***C. fleckeri*** **transcripts to** ***H. magnipapillata*** **and** ***N. vectensis*** **.** Bit scores from searches of the assembled transcripts against databases containing *H. magnipapillata* and *N. vectensis* proteins using blastx. Solid line is the best simple linear regression model which suggests that generally C. fleckeri protein products possess more sequence homology to H. magnipapillata than N. vectensis. Y = X is shown as a dotted line. Additional file 2:
**GO terms assigned to C. fleckeri transcripts.** Bar graph showing the number and percent of total of GO terms assigned to C. fleckeri transcripts using InterProScan. GO terms were reduced to level 2 terms and are grouped into the three components of the GO hierarchy; “Biological process”, “Cellular component” and “Molecular function”. Figure produced using WEGO [56]. Additional file 3:
**Summary of GO and InterProScan terms associated with transcripts.** Multi-worksheet excel file containing Go terms (Worksheet 1) and InterProScan terms (Worksheet 2) associated with *C. fleckeri* transcripts. Additional file 4
**Summary of toxins identified in the transcriptome of** ***C. fleckeri.*** Multi-worksheet excel file containing toxins identified in the transcript of *C. fleckeri* ranked by FPKM (Worksheet 1) and categorized by function (Worksheet 2). Additional file 5:
**Potential toxins identified in the proteome of** ***C. fleckeri*** **.** Excel file listing potential toxins identified in the venom proteome of *C. fleckeri* categorized by function. Additional file 6:
**Proteins identified in additional database searches.** Excel file listing proteins identified during additional database searches categorized by function. Additional file 7:
**X! Tandem protein reports.** Multi-worksheet excel file containing protein report for each search of *C. fleckeri* predicted protein set. Additional file 8:
**ProteinProphet report for OFFGEL/MS/MS experiments.** Excel file containing ProteinProphet report for searches using mass spectrometry data generated from OFFGEL experiments. Additional file 9:
**Mayu false discovery analysis of OFFGEL/MS/MS experiments.** Excel file containing Mayu false discovery analysis of searches using mass spectrometry data generated from OFFGEL experiments.

## Abbreviations

CfTX: *C. fleckeri* toxin
FPKM: Fragments per kilobase of exon per million fragments mapped
GO: Gene ontology
KEGG: Kyoto Encyclopedia of Genes and Genomes
OFFGEL: Offgel electrophoresis
