# Supplementary material for: Transcriptome and venom proteome of the box jellyfish Chironex fleckeri
Source: BMC Genomics. 2015 May 27;16(1):407. doi: 10.1186/s12864-015-1568-3 (PMC4445812; doi:10.1186/s12864-015-1568-3)
Supplement: Additional file 1: — Comparison of C. fleckeri transcripts to H. magnipapillata and N. vectensis . Bit scores from searches of the assembled transcripts against databases containing H. magnipapillata and N. vectensis proteins using blastx. Solid line is the best simple linear regression model which suggests that generally C. fleckeri protein products possess more sequence homology to H. magnipapillata than N. vectensis. Y = X is shown as a dotted line. [file 12864_2015_1568_MOESM1_ESM.pdf]

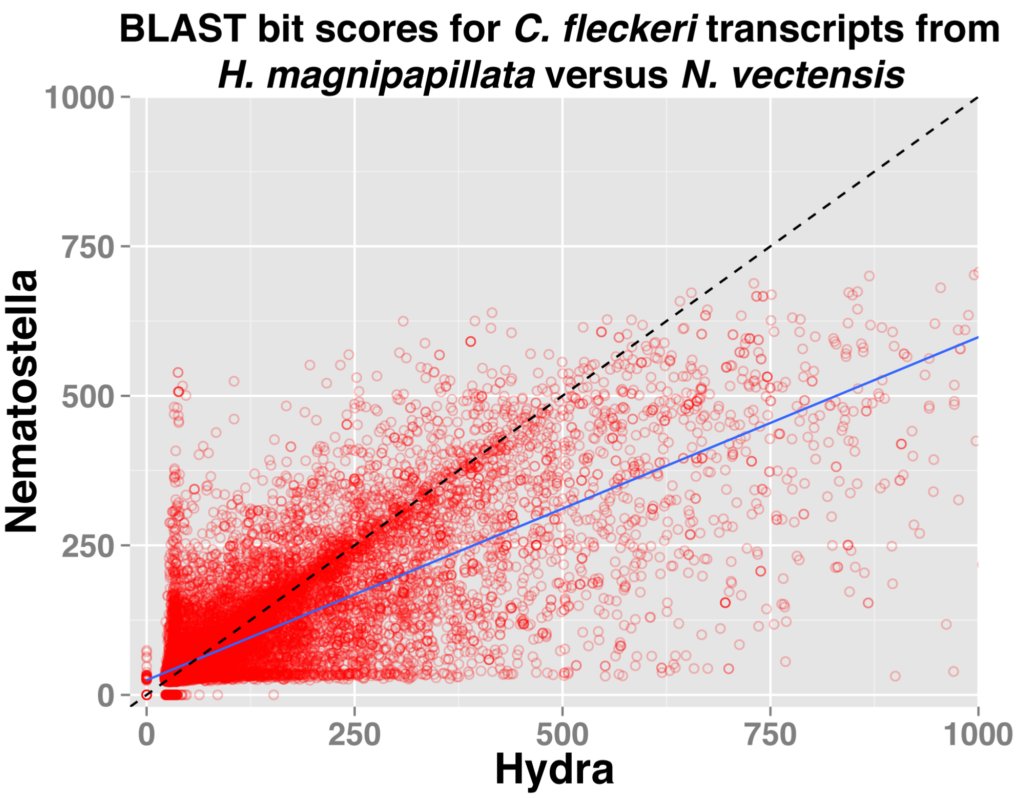

Supplementary Figure 1: Comparison of *C. fleckeri* transcripts to *H. magnipapillata* and *N. vectensis*. Bit scores from searches of the assembled transcripts against databases containing *H. magnipapillata* and *N. vectensis* proteins using blastx. Solid line is the best simple linear regression model which suggests that generally *C. fleckeri* protein products possess more sequence homology to *H. magnipapillata* than *N. vectensis*. Y = X is shown as a dotted line.
