# Supplementary material for: Transcriptome and venom proteome of the box jellyfish Chironex fleckeri
Source: BMC Genomics. 2015 May 27;16(1):407. doi: 10.1186/s12864-015-1568-3 (PMC4445812; doi:10.1186/s12864-015-1568-3)
Supplement: Additional file 2: — GO terms assigned to C. fleckeri transcripts. Bar graph showing the number and percent of total of GO terms assigned to C. fleckeri transcripts using InterProScan. GO terms were reduced to level 2 terms and are grouped into the three components of the GO hierarchy; “Biological process”, “Cellular component” and “Molecular function”. Figure produced using WEGO [56]. [file 12864_2015_1568_MOESM2_ESM.pdf]

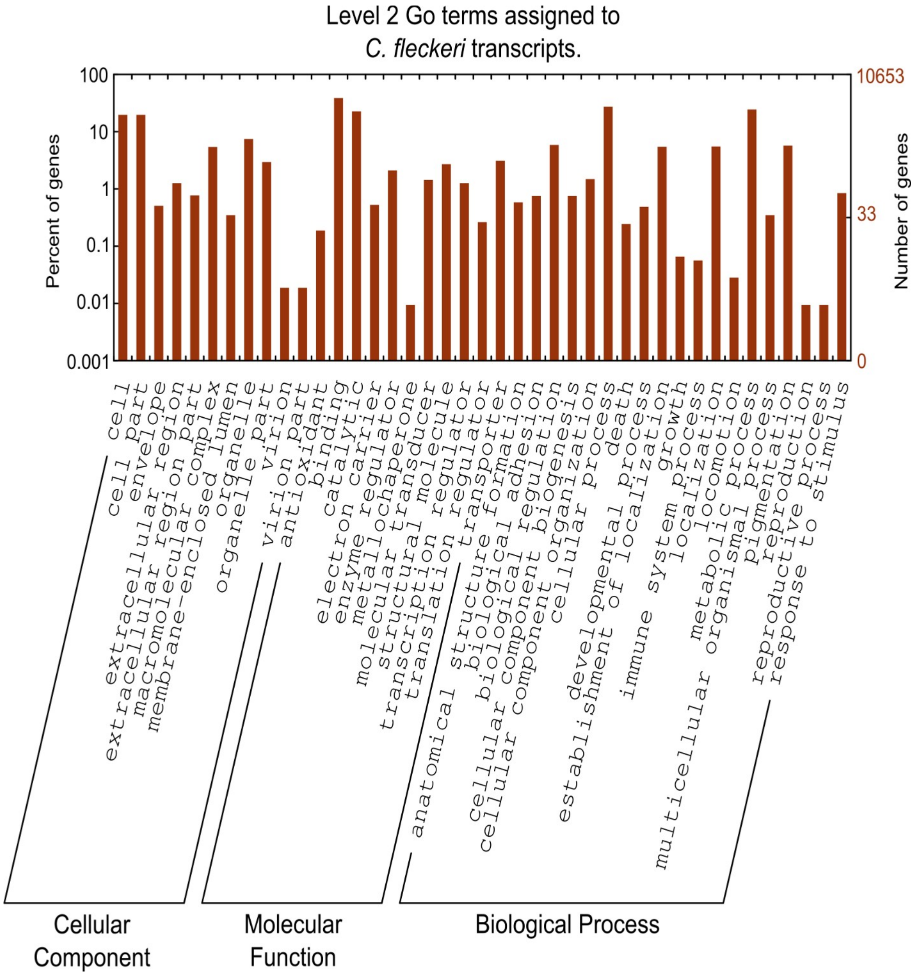

Supplementary Figure 2: GO terms assigned to *C. fleckeri* transcripts. Bar graph showing the number and percent of total of GO terms assigned to *C. fleckeri* transcripts using InterProScan. GO terms were reduced to level 2 terms and are grouped into the three components of the GO hierarchy; `Biological process', `Cellular component' and `Molecular function'. Figure produced using WEGO.
